# Extraction of First Molars

**Published:** 1880-05

**Authors:** A. W. Harlan

**Affiliations:** Chicago.


### ARTICLE V.

### *Extraction of First Molars.*

BY A. W. HARLAN, OF CHICAGO.

The diversity of opinion concerning the treatment of these teeth is so great, and their proper treatment under all circumstances so difficult to determine on the first visit of the patient, that many operators either hurriedly extract them, or are satisfied to as hurriedly fill them with inferior materials, thus hastening their ultimate destruction. I desire, therefore, to offer a few words with reference to the proper time of extracting them, and a few indications therefor.

First.—In all mouths where it is apparent that both jaws alike give evidence of being contracted from a normal-shaped arch, and the teeth are large and imperfectly developed, my rule would be to extract all four of them at the same time, even though only one of them should be seriously affected by caries.

Second.—In all cases where the patient is presented previous to the eleventh year with one pulpless molar of the above class, and the other three already so badly decayed that one or more pulps are exposed, I would extract, even if the shape of the arch were normal and no tendency existed to indicate that the teeth would be crowded or irregular in their final arrangement.

Third.—The rule is to extract when a patient is presented who has previously lost one of these teeth before the eleventh year, unless one of the bicuspids has also been extracted on the opposite side of the arch and not from the same maxilla. Then, perhaps, it would be better to extract the corresponding bicuspid; but if the articulation of the teeth were interfered with by so operating to any appreciable degree, the bicuspid should be extracted, and the remaining molar from the maxilla from whence the other had been extracted. Further, I would never extract a molar, or the molars of the sixth year, for the purpose of correcting an irregularity of the teeth anterior to them, if they were all alive and of reasonably dense structure.

Fourth.—The rule would be to extract the sixth year molars after the eruption of the second bicuspids in those cases where the teeth are large and were erupted early, and are of a chalky structure, from a perfectly normal arch, as these teeth invariably show caries on their distal and mesial surfaces, while there are no teeth in contact with them, often as early as the seventh to the eighth year, and it is next to impossible to save them. They should be extracted about the tenth year.

Fifth.—It is proper to extract what are known as honeycombed teeth, and those teeth showing arrested development as a result of exanthematous diseases, and also those teeth which bear the marks of inherited disease—meaning by the above descriptions teeth almost wholly devoid of shape and destitute of enamel, and already extensively decayed previous to the eighth or ninth year.

Conclusions. My observations of the mouths of children and adults, and study of authorities, lead me to conclude that in the above-named cases, the prospect for the patient having a perfect and regular arch is many times greater than the most skillful treatment by filling ever possibly produced. In such cases there is no interruption of the eruptive process of the twelfth year molar, and at the period of the eruption of the dentes sapientiæ, they come far forward of what would have been the case in the event of the non-extraction of the sixth year molars. It must be understood that I do not advise the indiscriminate extraction of these teeth ; you may see under what circumstances I do advocate their extraction.

This paper would be useless if the proper period for the extraction of the above teeth were not indicated. When we know that the molars were erupted at or about the sixth year, my rule is to extract not later than the tenth year; if they were erupted as late as the seventh year, I defer their extraction, if possible, until the eleventh year, and seldom later than that date.

One word more. It is claimed by some authorities that of late years a tendency has been observed by them indicating the total suppression of the third molars in the near future. Those who are fearful of that calamity will not view with favor the proposition to extract any of the above-named teeth, and they will undoubtedly continue to fill all such without reference to the future utility of a lesser number of teeth, or the ease and facility with which they may be cared for by patient and operator.—*Trans. of Ill State Dental Society.*

